# Multi-Modal AI for Multi-Label Retinal Disease Prediction Using OCT and Fundus Images: A Hybrid Approach

**DOI:** 10.3390/s25144492

**Published:** 2025-07-19

**Authors:** Amina Zedadra, Mahmoud Yassine Salah-Salah, Ouarda Zedadra, Antonio Guerrieri

**Affiliations:** 1 LabSTIC Laboratory, University 8 May 1945 Guelma, Algeria, BP 401, Guelma 24000, Algeria; zedadra.amina@univ-guelma.dz (A.Z.); zedadra.ouarda@univ-guelma.dz (O.Z.); 2 Medical-Surgical Ophthalmology Office, 284 CNEP Apartments, University Road, Block 37, No. 1, Guelma 24006, Algeria; dr.salahsalahophtalmo@gmail.com; 3 ICAR-CNR—Institute for High Performance Computing and Networking, National Research Council of Italy, Via P. Bucci 8/9C, 87036 Rende, Italy

**Keywords:** retinal image, ocular diseases, ophthalmology, Convolutional Neural Network (CNN), Large Language Model (LLM), Graph Neural Network (GNN)

## Abstract

Ocular diseases can significantly affect vision and overall quality of life, with diagnosis often being time-consuming and dependent on expert interpretation. While previous computer-aided diagnostic systems have focused primarily on medical imaging, this paper proposes VisionTrack, a multi-modal AI system for predicting multiple retinal diseases, including Diabetic Retinopathy (DR), Age-related Macular Degeneration (AMD), Diabetic Macular Edema (DME), drusen, Central Serous Retinopathy (CSR), and Macular Hole (MH), as well as normal cases. The proposed framework integrates a Convolutional Neural Network (CNN) for image-based feature extraction, a Graph Neural Network (GNN) to model complex relationships among clinical risk factors, and a Large Language Model (LLM) to process patient medical reports. By leveraging diverse data sources, VisionTrack improves prediction accuracy and offers a more comprehensive assessment of retinal health. Experimental results demonstrate the effectiveness of this hybrid system, highlighting its potential for early detection, risk assessment, and personalized ophthalmic care. Experiments were conducted using two publicly available datasets, RetinalOCT and RFMID, which provide diverse retinal imaging modalities: OCT images and fundus images, respectively. The proposed multi-modal AI system demonstrated strong performance in multi-label disease prediction. On the RetinalOCT dataset, the model achieved an accuracy of 0.980, F1-score of 0.979, recall of 0.978, and precision of 0.979. Similarly, on the RFMID dataset, it reached an accuracy of 0.989, F1-score of 0.881, recall of 0.866, and precision of 0.897. These results confirm the robustness, reliability, and generalization capability of the proposed approach across different imaging modalities.

## 1. Introduction

The retina, a vital structure located at the back of the eye, plays a fundamental role in vision by converting light signals into electrical impulses through photoreceptor cells, forming the basis of human visual perception. Owing to its delicate nature, the retina is particularly susceptible to a range of ocular diseases, including Diabetic Retinopathy (DR), Age-related Macular Degeneration (AMD), Diabetic Macular Edema (DME), drusen, Central Serous Retinopathy (CSR), and Macular Hole (MH). These conditions can progressively impair vision and severely affect a person’s quality of life if not detected and managed early.

According to the latest World Health Organization (WHO) report published in 2023, an estimated 2.2 billion people worldwide suffer from near- or distant-vision impairment. Among these, 94 million cases are due to cataracts, 7.7 million to glaucoma, 88.4 million to refractive errors, 3.9 million to Diabetic Retinopathy, and 8 million to Age-related Macular Degeneration [1]. The early and accurate diagnosis of retinal diseases is therefore crucial to preventing severe visual impairment and blindness.

In recent years, Artificial Intelligence (AI) has emerged as a transformative tool in the field of medical imaging (e.g., [2,3]), offering the potential to automate the analysis of different imaging modalities such as fundus photography and Optical Coherence Tomography (OCT). These AI-driven systems have proven capable of supporting ophthalmologists in accurately diagnosing retinal conditions, reducing diagnostic workload, and improving screening efficiency. Numerous studies have employed advanced deep learning and machine learning models for disease detection, achieving high diagnostic accuracy and, in many cases, surpassing traditional manual methods [1,4,5,6,7].

However, while much of the existing research focuses on classifying and detecting ocular diseases using medical imaging alone, limited work has addressed multi-label disease prediction or incorporated clinical risk factors and textual patient data. Furthermore, predictive models capable of forecasting the progression of these diseases over time remain underexplored. Developing such predictive frameworks could significantly improve early intervention strategies and personalized treatment planning, ultimately reducing the risk of vision loss.

To address this gap, this study proposes VisionTrack, a novel multi-modal AI framework designed for multi-label ocular disease prediction. VisionTrack integrates a Convolutional Neural Network (CNN) to extract discriminative features from retinal images, a Graph Convolutional Network (GCN) to capture complex interrelations among key clinical risk factors such as age, diabetes status, hypertension, and disease duration, and a Large Language Model (LLM) to analyze unstructured textual data from patient medical reports. By effectively combining these heterogeneous data sources, VisionTrack improves predictive accuracy, enables the simultaneous prediction of multiple ocular conditions, and provides a more holistic and personalized assessment of retinal health.

The remainder of this paper is organized as follows: Section 2 reviews the related work on retinal diseases, including dedicated subsections for Age-related Macular Degeneration (Section 2.1), Diabetic Retinopathy (Section 2.2), Diabetic Macular Edema (Section 2.3), drusen (Section 2.4), Central Serous Retinopathy (Section 2.5), and Macular Hole (Section 2.6). Section 3 presents the proposed multi-modal AI framework, while Section 4 describes the experimental setup and the datasets used and discusses the performance of the proposed method in comparison with state-of-the-art techniques. Finally, Section 5 concludes the paper and outlines future research directions.

## 2. Background

We provide a comprehensive review of existing approaches for disease prediction using medical images and the application of advanced AI techniques for six major retinal diseases: Diabetic Retinopathy (DR), Age-related Macular Degeneration (AMD), Diabetic Macular Edema (DME), drusen, Central Serous Retinopathy (CSR), and Macular Hole (MH).

### 2.1. Age-Related Macular Degeneration

Age-related Macular Degeneration (AMD) is a major cause of vision loss worldwide, necessitating early and precise diagnosis. Advances in Artificial Intelligence (AI), particularly deep learning and machine learning, have led to substantial improvements in AMD detection using fundus images and Optical Coherence Tomography (OCT). This paper summarizes key studies that employ AI methodologies for diagnosing AMD, analyzing their objectives, datasets, and outcomes. The reviewed studies utilize Convolutional Neural Networks (CNNs), Vision Transformers (ViTs), and hybrid AI models to enhance classification accuracy and interpretability. The results demonstrate promising performance, achieving high sensitivity, specificity, and accuracy, underscoring AI’s potential in ophthalmology.

Several studies have explored AI-driven approaches for AMD detection. El-Sharkawy et al. [8] developed an explainable AI system for AMD grading using OCT images, achieving 90.82% accuracy in a multi-way classification task. Abd El-Khalek et al. [9] introduced a novel ML-based classification framework for AMD diagnosis from fundus images, achieving 96.85% accuracy. Yang et al. [10] proposed an ensemble deep learning model integrating multiple CNN architectures for dry AMD classification, significantly improving accuracy. Le et al. [11] designed ViT-AMD, a Vision Transformer-based model for AMD diagnosis, achieving superior performance compared to CNN-based models. Chen et al. [12] developed a deep learning model to generate ICGA images from fundus photographs using GANs, enhancing AMD classification accuracy. Table 1 summarizes the related work introduced in this section.

### 2.2. Diabetic Retinopathy

Diabetic Retinopathy (DR) is a leading cause of vision impairment globally, necessitating early and precise diagnosis. Advances in Artificial Intelligence (AI), particularly deep learning and machine learning, have significantly improved DR detection using fundus images and Optical Coherence Tomography (OCT). This paper summarizes key studies that employ AI methodologies for diagnosing DR, analyzing their objectives, datasets, and outcomes. The reviewed studies utilize Convolutional Neural Networks (CNNs), Vision Transformers (ViTs), Graph Neural Networks (GNNs), and hybrid AI models to enhance classification accuracy and interpretability. The results demonstrate promising performance, achieving high sensitivity, specificity, and accuracy, underscoring AI’s potential in ophthalmology.

Several studies have explored AI-driven approaches for DR detection. Akram et al. [13] developed a Bayesian deep learning model integrating Monte Carlo Dropout and Variational Inference to enhance uncertainty estimation, achieving 97.68% accuracy on the APTOS 2019 and DDR datasets. Liu et al. [14] introduced a Vision Transformer model incorporating softmax-pooling operators, outperforming CNN-based models in DR grading. Wong et al. [15] proposed a transfer learning approach combining ShuffleNet and ResNet-18 with an ECOC ensemble, reaching 96% accuracy in binary classification. Maaliw et al. [16] presented a segmentation-based DR detection system using DR-UNet and an attention-aware CNN, achieving a high accuracy of 99.2% on DiaretDB0 and DiaretDB1 datasets. In a recent study, Zedadra et al. [17] demonstrated the value of using complementary data alongside images to improve classification performance. Specifically, they introduced a risk factor: the duration of diabetes. By employing a model that combines a Convolutional Neural Network (CNN) with a Graph Neural Network (GNN), they achieved very promising results. Sumod and Sumathy [18] developed a graph-based AI model for DR grading using fundus images, achieving superior performance in feature extraction. Poranki and Rao [19] introduced an XGBoost-based classification framework for DR diagnosis, attaining 99.6% accuracy. Zhang et al. [20] proposed a multi-model domain adaptation approach for unsupervised DR classification, showing improved generalizability. Feng et al. [21] designed a GNN-CNN hybrid model for DR grading, significantly enhancing feature relationship capture. Shamrat et al. [22] developed DRNet13, a CNN-based model that outperformed traditional architectures with 97% accuracy. Dhinakaran et al. [23] introduced a semi-supervised graph learning method to handle imbalanced datasets, improving DR risk prediction. Table 2 summarizes the related work introduced in this section.

### 2.3. Diabetic Macular Edema

The early detection of DME is essential, as it can prevent over 95% of vision loss associated with the condition or at least slow its progression through timely interventions. DME is marked by various abnormalities in the retinal vasculature, such as retinal edema, hard exudates, retinal hemorrhages, and intraretinal microvascular anomalies [24].

Several studies have explored AI-driven approaches for Diabetic Macular Edema (DME) detection. Fu [24] introduced the Multi-feature Decomposition Fusion Attention Network (MDFANet) for DME classification in multicolor imaging. The model integrates a Lite Transformer and an Invertible Neural Network to capture multi-frequency features, improving detection accuracy. Zhang et al. [25] developed a deep learning model using ultra-widefield fundus imaging for referable DR and DME detection. Their approach, based on EfficientNet and ResNet, demonstrated robust performance in large-scale automated screening.

Wu et al. [26] designed a deep learning framework to detect morphological patterns of DME from OCT images. Their model achieved high accuracy in identifying diffused retinal thickening, cystoid macular edema, and serous retinal detachment. Saidi et al. [27] implemented a CNN-based model for the automatic detection of AMD and DME, obtaining over 99% accuracy on the Duke dataset. Tripathi et al. [28] proposed a GAN-based model to generate synthetic OCT B-Scan images for DME detection. By leveraging StyleGAN and CycleGAN, they improved the robustness of automated diagnostic models. Thanikachalam et al. [29] developed an optimized deep CNN for DR and DME classification, incorporating adaptive Gabor filters and Random Forest-based feature selection. Their method achieved 97.91% accuracy on benchmark datasets. Nazir et al. [30] introduced a CenterNet-based deep learning model for DME detection from retinal images. By using DenseNet-100 for feature extraction, their system outperformed traditional CNN architectures. Table 3 summarizes the related work introduced in this section.

### 2.4. Drusen

Recent advances in AI-based drusen detection have focused on segmentation, classification, and the grading of drusen patterns in retinal images. Goyanes et al. [31] introduced a fully automatic 3D deep learning-based segmentation method for detecting drusen in OCT images, significantly improving diagnostic workflows. Omar et al. [32] introduced a classification approach using bagged color vector angles for exudate and drusen differentiation. Ilyasova et al. [33] focused on the recognition of drusen subtypes in OCT images for diagnosing Age-related Macular Degeneration (AMD). The authors proposed a segmentation-based method to extract drusen from images and classify them based on reflectivity features. Nowomiejska et al. [34] proposed a Residual Attention Network to distinguish optic disc drusen from healthy optic discs. Table 4 summarizes the related work introduced in this section.

### 2.5. Central Serous Retinopathy

Central Serous Retinopathy (CSR), also known as Central Serous Chorioretinopathy (CSC), is a serious eye condition that affects millions of people globally, often leading to vision loss or even blindness. It occurs due to the buildup of fluid beneath the retina. Early detection is crucial, as it enables timely intervention to prevent lasting damage to vision. While traditional manual diagnostic methods have been used, they often lack accuracy and reliability. As a result, advancements in Artificial Intelligence have paved the way for more effective and automated approaches to CSR detection and treatment Hassan et al. [35].

Recent advances in the AI-based detection of CSR have focused on automated diagnosis using fundus and OCT images and distinguishing between acute and chronic CSC subtypes. Zhen [36] proposed a deep learning method based on the InceptionV3 model for classifying CSC from color fundus photographs. Their system outperformed human ophthalmologists in terms of accuracy and agreement scores. Yoon et al. [37] introduced a deep learning system trained on spectral-domain Optical Coherence Tomography (SD-OCT) images to diagnose CSC and differentiate between acute and chronic forms. The model showed high diagnostic accuracy and performance comparable to or superior to that of expert clinicians. Hassan et al. [38] presented a comprehensive framework using modified DenseNet and DarkNet classifiers trained on OCT and fundus images, achieving state-of-the-art performance in CSR detection. The work in [39] proposed a deep learning approach using ResNet50 CNNs on multiple OCT modalities to predict the 6-month persistence of CSC, achieving up to 95.2% accuracy by combining B-scan and retinal thickness images.

Table 5 summarizes these AI-based approaches.

### 2.6. Macular Hole

Macular Hole (MH) is a serious retinal disorder that compromises central vision and requires timely, accurate diagnosis for effective treatment. With an estimated prevalence of 7 per 100,000 individuals globally, the need for reliable diagnostic tools is critical. This study aims to develop an AI model capable of accurately differentiating MH from normal cases using Optical Coherence Tomography (OCT) scans. The goal is to provide ophthalmologists with a fast, precise diagnostic aid that facilitates early detection and enhances clinical decision-making and patient outcomes Bolanos et al. [39].

Recent advances in the AI-based detection of Macular Hole (MH) have explored both segmentation and classification techniques using Optical Coherence Tomography (OCT) images. Shahalinejad [40] proposed a hybrid method combining multilevel thresholding and derivation-based edge detection for MH diagnosis from OCT images. Their technique demonstrated improved sensitivity and accuracy over conventional image processing methods. More recently, Ko [41] introduced a deep learning-based multi-image classification model for assessing central serous chorioretinopathy (CSC), achieving expert-level performance in distinguishing acute, chronic, and normal cases from multiple SD-OCT images. While their focus was CSC, the multi-scan ensemble architecture has direct implications for future AI-based MH detection systems.

Table 6 summarizes these AI-based approaches.

## 3. Hybrid Multi-Modal Eye Disease Prediction System

**Figure 1:**
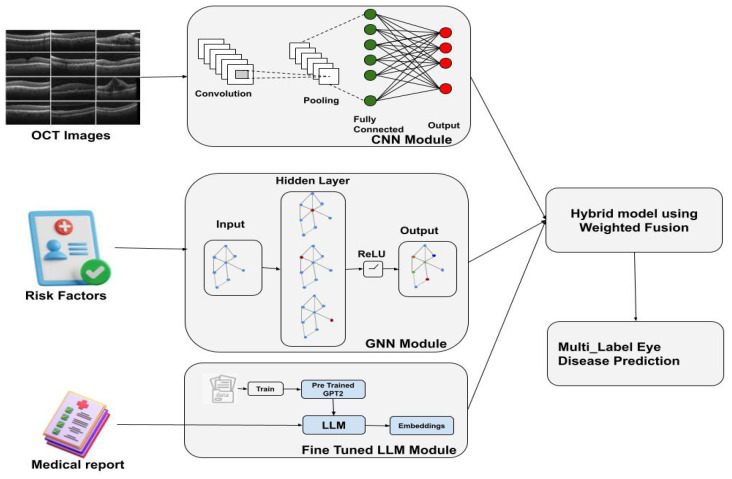
System architecture of the proposed hybrid multi-modal eye disease prediction model.

The proposed approach is a hybrid multi-modal model designed to predict multiple eye diseases simultaneously (Figure 1). It focuses on six common conditions: **Diabetic Macular Edema (DME), Diabetic Retinopathy (DR), Age-related Macular Degeneration (AMD), Central Serous Retinopathy (CSR), Macular Hole (MH), and drusen**. The system integrates information from three sources:

- A **Convolutional Neural Network (CNN)** for feature extraction from retinal images.
- A **Graph Neural Network (GNN)** for processing patient metadata and risk factors.
- A **Large Language Model (LLM)** for analyzing unstructured clinical text from medical reports.

### 3.1. Data Preprocessing

The proposed approach, as the first step, involves some data preprocessing, which has to be carried out in order to optimize input sequences to the proposed hybrid model. In particular, this involves the following:

- **Retinal Images**: These images are preprocessed through a series of steps to normalize and augment the data. The transformations include resizing the images to a standard size, normalization based on mean and standard deviation values (such as μ=[0.485,0.456,0.406]; σ=[0.229,0.224,0.225]), and data augmentation techniques like random horizontal flipping, rotation, and brightness adjustments to increase data diversity and reduce overfitting.
- **Risk Factor Metadata**: Clinical features such as age, hypertension (HTA), diabetes (diabetic status, duration), smoking, and dyslipidemia are preprocessed by normalizing the values into numerical vectors. Each clinical feature $x_{i}$ is normalized as follows:
  (1) $$x_{i}^{\prime }=\frac{x_{i}-\mu_{i}}{\sigma_{i}}$$
  where $\mu_{i}$ and $\sigma_{i}$ are the mean and standard deviation of the feature $x_{i}$ in the dataset.
- **Medical Reports**: The unstructured text data from medical reports is preprocessed using natural language processing (NLP) techniques, including tokenization, stopword removal, and vectorization (e.g., using word embeddings or transformers) to convert the text into numerical features that can be used by the model.

### 3.2. Model Architecture

**Figure 2:**
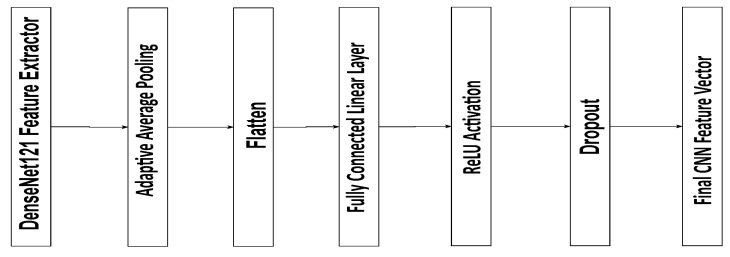
The proposed CNN-based feature extraction.

The model consists of three main components, each processing different modalities:

- **Convolutional Neural Network (CNN) for Retinal Image Feature Extraction**: The CNN extracts features from retinal images, typically using a pre-trained backbone such as DenseNet or ResNet. The input image *I* is passed through several convolutional layers to obtain a high-level representation $f_{I}$:
  (2) $$f_{I}=\mathrm{CNN}\left(I\right)$$
  The output of the CNN is then passed through fully connected layers to obtain a final feature vector, $f_{\mathrm{retinal}}$, representing the image’s characteristics.
  The CNN module in the proposed model is based on a pre-trained DenseNet121 architecture used for feature extraction from retinal images. The extracted feature maps are first processed through an Adaptive Average Pooling layer, reducing their spatial dimensions to a single value per channel. The resulting pooled features are then flattened into a one-dimensional vector of the size 1024. This vector passes through a fully connected linear layer that preserves the 1024 feature dimensions, followed by a ReLU activation to introduce non-linearity and a Dropout layer with a 0.5 probability to prevent overfitting. The final output is a 1024-dimensional feature vector representing the high-level image features, which is subsequently used for disease classification and integrated into the multi-modal fusion process of the model (Figure 2).
- **Graph Neural Network (GNN) for Clinical Data Integration**: The clinical features, including risk factors like age, hypertension, and smoking, are encoded as node features in a graph. Each patient is represented as a node, and edges are created based on relationships between patients, such as shared disease history or similar demographics. The node features are updated through graph convolutions. The graph convolution operation is given by
  (3) $$h^{\left(l+1\right)}=\sigma \left(\hat{A}h^{\left(l\right)}W^{\left(l\right)}\right)$$
  where $\hat{A}$ is the normalized adjacency matrix of the graph, $h^{\left(l\right)}$ is the feature matrix in layer *l*, $W^{\left(l\right)}$ is the learned weight matrix, and σ is the activation function (e.g., ReLU or SiLU).
  The final output $f_{\mathrm{gnn}}$ from the GNN is a feature representation of the clinical data, which is combined with the retinal image features.
  The GNN module receives input node features formed by concatenating the 1024-dimensional CNN feature vectors, four binary clinical variables, and embedded continuous features for age and diabetes duration. These combined features are passed through a first GCNConv layer that projects them to 256 dimensions, followed by Batch Normalization, ReLU activation, and Dropout for regularization. A second GCNConv layer then expands the feature dimension to 1024. Finally, a global mean pooling operation aggregates the node-level features into a single 1024-dimensional vector, representing the integrated image-clinical profile for disease prediction.
- **Large Language Model (LLM) for Medical Report Analysis**: The LLM processes the unstructured clinical text data (e.g., physicians’ reports) to extract contextual information relevant to the eye disease prediction. The text data is tokenized and passed through the model to generate embeddings that capture the semantic meaning of the reports. These embeddings are represented as $f_{\mathrm{text}}$:
  (4) $$f_{\mathrm{text}}=\mathrm{LLM}\left(text\right)$$
  These embeddings are integrated with the retinal and clinical features in the final decision-making stage.

### 3.3. Fusion and Prediction

The features obtained from the three modalities—retinal images, clinical metadata, and medical reports—are fused together into a single representation, $f_{\mathrm{fusion}}$:

(5) $$f_{\mathrm{fusion}}=\mathrm{concat}\left(f_{\mathrm{retinal}},f_{\mathrm{gnn}},f_{\mathrm{text}}\right)$$

This fused representation is passed through a series of fully connected layers to output a prediction vector:

(6) $$\hat{y}=\sigma \left(Wf_{\mathrm{fusion}}+b\right)$$

where *W* is the weight matrix, *b* is the bias, and σ is the sigmoid activation function. The final prediction $\hat{y}$ gives the probability of each of the diseases in the multi-class setting.

### 3.4. Medical Rule Application

To enhance the clinical validity of the prediction system, a set of expert-defined clinical rules was applied as a postprocessing step. These rules were established in collaboration with an experienced ophthalmologist based on current clinical practice guidelines and epidemiological evidence. The rules adjust the raw multi-label prediction vector $\hat{y}$ based on patient-specific metadata (e.g., age, diabetes status, hypertension) to eliminate medically implausible predictions and improve clinical coherence.

The adjusted predictions are computed as follows:

(7) $${\hat{y}}^{\mathrm{adjusted}}=\hat{y}\;\cdot \;R$$

where *R* is a rule-based binary mask vector derived from patient clinical metadata and the expert clinical rules listed below.

Each element of *R* is set to 0 if the corresponding disease prediction should be suppressed based on the clinical context and 1 otherwise.

This adjustment step ensures that the final model outputs remain consistent with medical knowledge and real-world clinical expectations.

The following clinical rules were defined and validated by an ophthalmologist for use in this study:

- If Diabetes = No, set Diabetic Retinopathy (DR) and Diabetic Macular Edema (DME) predictions to 0.
- If Age < 50, set Age-related Macular Degeneration (AMD) prediction to 0.
- If Hypertension = No, reduce the risk prediction for Retinal Vein Occlusion (RVO)-related diseases.
- If Smoking History = No, reduce the risk prediction for AMD and Central Serous Retinopathy (CSR).
- If Duration of Diabetes < 5 years, suppress severe DR grade predictions.

## 4. Experiments

### 4.1. Dataset

We utilized two distinct datasets: the RFMiD and the Retinal OCT dataset. The former comprises fundus images, while the latter includes both fundus and OCT images, providing diverse modalities for comprehensive retinal disease analysis. Furthermore, we selected a subset of seven categories (DME, DR, AMD, CSR, MH, drusen, and normal) from both the RFMID and Retinal OCT dataset to address potential class imbalance issues and focus on the most prevalent and clinically significant retinal conditions.

#### 4.1.1. RFMID Dataset

The Retinal Fundus Multi-disease Image Dataset (RFMiD) comprises 3200 fundus images acquired using three distinct fundus cameras. Each image is annotated with one or more of 46 retinal conditions, based on the adjudicated consensus of two experienced retinal specialists [42]. The dataset is split into three subsets as suggested by the dataset creators: 60% (1920 images) for training, 20% (640 images) for evaluation, and 20% (640 images) for testing. The fundus images were captured using non-invasive fundus cameras while the patient was seated upright. The distance between the lenses and the examined eye was maintained at 39 mm for the Kowa VX-10 (Kowa Company, Ltd., Nagoya, Japan) and 40.7 mm for the TOPCON 3D OCT-2000 and TOPCON TRC-NW300 devices (Topcon Positioning Systems, Inc., Tokyo, Japan). The dataset includes images acquired with three camera models: the Nikon D7000 (Nikon Corporation, Tokyo, Janpan) digital camera (used with the TOPCON 3D OCT-2000), the Nikon D70s digital camera (used with the Kowa VX-10α), and an integrated digital CCD camera (used with the TOPCON TRC-NW300). The corresponding image resolutions are 2144 × 1424 pixels for TOPCON 3D OCT-2000, 4288 × 2848 pixels for Kowa VX-10α, and 2048 × 1536 pixels for TOPCON TRC-NW300. The field of view (FOV) ranges from 45° to 50°, and both high-quality and low-quality images are deliberately included to increase the dataset’s complexity and reflect real-world variability.

#### 4.1.2. Retinal OCT Dataset

The Retinal OCT-8 Classes dataset comprises 24,000 Optical Coherence Tomography (OCT) images, each labeled across eight distinct retinal disease categories. It is designed to support research and model training in retinal disease classification using machine learning and deep learning techniques [43]. The dataset is also split according to the recommendations of its creators: 75% for training (18,400 images), 15% for validation (2800 images), and 15% for testing (2800 images). The images in the OCT dataset are of varying sizes but are consistently provided in JPEG format.

### 4.2. Evaluation Settings

Through extensive experimentation, we carefully tuned the hyperparameters to achieve optimal performance, balancing convergence speed, stability, and generalization while minimizing overfitting. We employed a systematic validation process, including k-fold cross-validation (with k = 5) and Bayesian optimization (via Optuna), to identify the best hyperparameters for our models.

For both the RFMiD (fundus images) and Retinal OCT (OCT images) dataset, we explored a range of learning rates, including 0.0005, 0.0002, 0.0001, 0.005, 0.002, 0.001, and 0.01. After extensive testing, a learning rate of 0.0001 was selected, as it provided the most favorable balance between convergence speed, training stability, and generalization. The number of training epochs was systematically evaluated within a range from 1 to 100, and we determined that 30 epochs achieved optimal performance without signs of overfitting. In addition, batch sizes of 16, 32, and 64 were tested, with 32 being chosen as the ideal value for balancing model generalization, training efficiency, and memory usage.

The hyperparameters explored and the selected values are summarized in Table 7.

### 4.3. Results

To evaluate the performance of the model, we use four metrics, including the following:

- **Accuracy**: The proportion of correctly predicted labels out of all predictions.
  (8) $$\mathrm{Accuracy}=\frac{\sum_{i=1}^{N}⊮\left({\hat{y}}_{i}=y_{i}\right)}{N}$$
  where ⊮ is the indicator function that equals 1 when the predicted label is correct.
- **Precision, Recall, and F1-Score**: These metrics are calculated for each disease class and averaged to provide an overall performance measure. The precision, recall, and F1-score for each class are computed as follows:
  (9) $$\mathrm{Precision}=\frac{\mathrm{TP}}{\mathrm{TP}+\mathrm{FP}},\;\mathrm{Recall}=\frac{\mathrm{TP}}{\mathrm{TP}+\mathrm{FN}},\;F1=2\times \frac{\mathrm{Precision}\times \mathrm{Recall}}{\mathrm{Precision}+\mathrm{Recall}}$$
  where TP, FP, and FN represent the true positives, false positives, and false negatives, respectively.

#### 4.3.1. Comparison with the Baseline Models

The performance comparison results presented in Table 8 and Table 9 highlight the effectiveness of the proposed VisionTrack model relative to a range of established baseline models. On the RFMiD dataset (Table 8), VisionTrack significantly outperformed all other models across every evaluation metric. While the best baseline models, MobileNet and GoogleNet, achieved an accuracy of 0.710 and F1-scores of approximately 0.518 and 0.517, respectively, VisionTrack obtained a markedly higher accuracy of 0.989 and an F1-score of 0.881. Similarly, VisionTrack recorded superior precision (0.897) and recall (0.866), exceeding the highest baseline precision (0.679 with MobileNet) and recall (0.472 with DenseNet121).

On the Retinal OCT dataset (Table 9), although the baseline models performed relatively well, with accuracies ranging from 0.960 to 0.970 and F1-scores between 0.960 and 0.972, VisionTrack still demonstrated the highest performance across all metrics. Specifically, VisionTrack achieved an accuracy of 0.980, a precision of 0.979, a recall of 0.978, and an F1-score of 0.979. Notably, DenseNet121 was the strongest baseline with an accuracy of 0.970 and an F1-score of 0.967, yet it remained slightly behind VisionTrack. These consistent improvements across two diverse ophthalmic datasets confirm the robustness, reliability, and generalizability of the VisionTrack architecture in comparison to conventional CNN and Transformer-based models.

**Figure 3:**
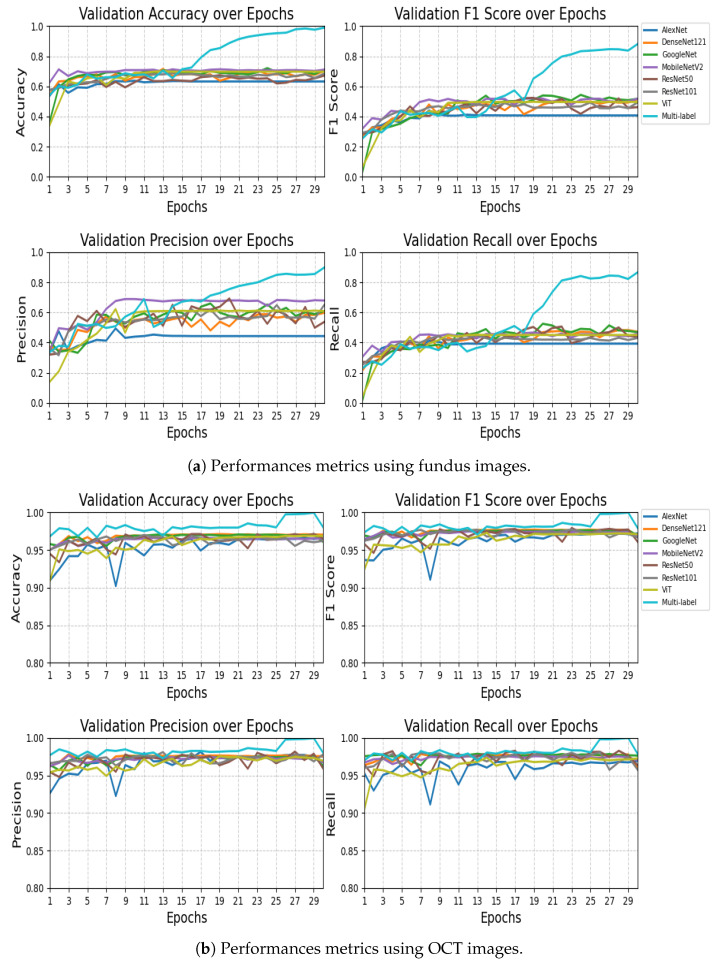
Performance metrics of the proposed model: (**a**) using fundus images and (**b**) using OCT images.

Figure 3 presents the results of the proposed model using fundus images (Figure 3a) and using OCT images (Figure 3b).

#### 4.3.2. Comparison with Other State-of-the-Art Methods

Table 10 compares the VisionTrack with other methods from the state of the art.

#### 4.3.3. Discussion

The experimental results presented in Table 10 demonstrate the clear superiority of VisionTrack over existing state-of-the-art methods across both the RFMiD and Retinal OCT dataset. Firstly, VisionTrack consistently outperformed other approaches in terms of **accuracy**, achieving 0.989 on the RFMiD and 0.980 on the Retinal OCT dataset. This performance indicates its strong capability to correctly classify a wide range of ocular diseases, surpassing previous leading models such as DFex-BeeHive [44] and DSAN-PL [46]. In addition to accuracy, VisionTrack exhibited superior **specificity**, notably reaching 0.999 on the RFMiD and 0.997 on the Retinal OCT dataset. High specificity is crucial in medical diagnosis, as it minimizes false positive rates, reducing the likelihood of healthy individuals being incorrectly diagnosed with eye diseases. This performance illustrates VisionTrack’s capacity to reliably exclude non-diseased cases, enhancing clinical trust in its predictions. Moreover, the framework achieved an **F1-score** of 0.881 on the RFMiD and 0.979 on the Retinal OCT dataset, outperforming competing methods. The F1-score, which balances precision and recall, confirms VisionTrack’s ability to maintain both a high true positive rate and precision, ensuring both sensitivity to actual disease cases and robustness against over-predicting positive results. A key factor contributing to these outcomes is the **multi-modal architecture** of VisionTrack, which integrates CNN-based image analysis, the GNN-based relational modeling of patient clinical data, and the LLM-driven interpretation of diagnostic reports. This combination allows the system to capture complementary patterns from heterogeneous data sources, providing a richer and more contextualized understanding of each patient case compared to unimodal models.

This study is limited by the use of the Retinal OCT-8 dataset, which is of low quality, with very little known about its provenance, particularly regarding image augmentation and the potential risk of data leakage between training and test sets.

### 4.4. Statistical Tests

To evaluate whether the performance differences between the proposed VisionTrack model and the baseline models were statistically significant, we conducted appropriate statistical tests. Specifically, we compared model performance across multiple runs using paired t-tests on key metrics: accuracy, F1-score, recall, and precision.

#### 4.4.1. Statistical Tests for Multi-Label Fundus Model

Table 11 presents the paired *t*-test results comparing Multi-label Fundus against other models.

The statistical analysis reveals that Multi-label Fundus significantly outperformed all baseline models across every metric, with extreme significance values ranging from $p=2.8\times 10^{-13}$ to $p=1.5\times 10^{-16}$. The performance advantage was particularly notable in accuracy (26.81% to 65.37% improvement) and F1-score (34.38% to 62.63% improvement), demonstrating the framework’s superior classification capabilities. While MobileNetV2 achieved the highest precision among baseline models (0.6889), it still fell substantially short of Multi-label Fundus’s 0.8976 precision ($p=9.4\times 10^{-16}$). The consistently extreme significance values (21/28 tests showing $p<1\times 10^{-15}$) provide overwhelming evidence that Multi-label Fundus represents a major advancement over conventional architectures for this task. These results validate the exceptional effectiveness of the Multi-label Fundus approach, which maintained robust performance across all evaluation metrics while traditional models exhibited limitations in either precision or recall.

#### 4.4.2. Statistical Tests for Multi-Label OCT Model

Table 12 presents the paired *t*-test results comparing Multi-label OCT against other models.

The statistical analysis demonstrates that Multi-label OCT achieved significantly superior performance compared to all baseline models, with extreme significance values ($p<1\times 10^{-13}$) across all metrics. The performance gap was most dramatic in the final epochs, where Multi-label OCT reached near-perfect accuracy (0.9997) compared to the best baseline (DenseNet121 at 0.9709). This represents an absolute improvement of 2.88% in accuracy and 2.23% in F1-score over the strongest conventional architecture. The precision advantage was particularly noteworthy, with Multi-label OCT achieving 0.9997 precision versus 0.9811 for ResNet101 ($p=4.5\times 10^{-16}$). All 28 statistical tests showed *p*-values below 1 × 10^−13^, with 24/28 below 1 × 10^−15^, providing overwhelming evidence for the superiority of the Multi-label OCT approach. The model’s exceptional performance is consistent across all metrics, avoiding the precision-recall tradeoffs observed in baseline models (e.g., Vision Transformer’s 2.15% lower recall compared to its precision). These results validate Multi-label OCT as a substantial advancement in ophthalmic image analysis, particularly in its ability to maintain robust performance throughout all training epochs while conventional models plateau earlier.

## 5. Conclusions and Future Work

Retinal diseases remain among the leading causes of vision loss worldwide, with many patients presenting multiple co-occurring conditions in real-world clinical practice. Although existing computer-aided diagnostic systems have shown promising results, most are limited to detecting individual diseases such as Diabetic Retinopathy (DR) or Age-Related Macular Degeneration (AMD). Moreover, challenges such as the long-tailed distribution of disease cases and frequent label co-occurrence complicate accurate and reliable diagnosis [25].

To address these limitations, this study proposed a multi-modal AI framework that integrates a Convolutional Neural Network (CNN) for OCT image feature extraction, a Graph Neural Network (GNN) for modeling complex relationships among clinical risk factors (including hypertension, diabetes duration, dyslipidemia, smoking status, and age), and a Large Language Models (LLM) for analyzing patient medical reports. This hybrid system facilitates multi-label prediction of retinal diseases such as DME, DR, AMD, drusen, CSR and MH, offering a more comprehensive and accurate assessment of retinal health.

Experimental evaluations demonstrated that the proposed framework outperformed existing methods, achieving high accuracy, F1-scores, recall, precision, and specificity across two public datasets. These results confirm the robustness and clinical applicability of the approach, providing an interpretable and effective solution for early detection and personalized ophthalmic care.

Looking ahead, future work will aim to enhance model performance by incorporating larger and more diverse datasets, conducting real-time clinical validation, and further optimizing the system’s ability to predict disease progression over time. Additionally, we envisage extending this approach to integrate multi-modal imaging data, combining both fundus photographs and OCT images, to improve diagnostic precision and capture complementary disease-related information. These advancements could pave the way for more effective early intervention strategies and precision medicine in ophthalmology. Also, we plan to integrate VisionTrack into clinical workflows by enabling real-time analysis directly from retinography devices that provide fundus/OCT images and clinical data, with results presented through automatically generated medical reports to support timely and informed decision-making.

## Figures and Tables

**Table 1:** Summary of AI-based AMD detection studies.

Reference	Objective	AI Tool	Used Dataset(s)	Images Analyzed	Obtained Results
[8]	AMD grading	CNN	1285 OCT B-scans	OCT images	Accuracy = 90.82%
[9]	AMD diagnosis	LightGBM (LGBM), Histogram-based Gradient Boosting (HGB), XG- Boost (XGB), AdaBoost, Random Forest (RF), Multi-Layer Perceptron (MLP), Decision Tree (DT), logistic regression (LR), support vector machine (SVM), and K-nearest neighbor (KNN)	-	Fundus images	Accuracy = 96.85%; Sensitivity = 93.72%; Specificity = 97.89%; Precision = 93.86%
[10]	Dry AMD classification	CNN	1310 patients (16,384 images)	OCT images	Accuracy (normal = 96.66%; drusen = 94.85%; nGA = 98.21%; GA = 96.31%)
[11]	AMD diagnosis	Vision Transformer (ViT)	Chula-AMD dataset	Fundus images	Accuracy = 93.40%; Sensitivity = 91.27%; Specificity = 96.57%
[12]	Age-related Macular Degeneration screening	Generative Adversarial Networks (GANs)	1172 patients (3195 CF images and 53,264 ICGA images)	Color fundus photography (CF) and CF+ translated indocyanine green angiography (ICGA)	F1-score = 0.88; Sensitivity = 0.88; Specificity = 0.94; Accuracy = 0.88; AUC = 0.96

**Table 2:** Summary of AI-based DR detection studies.

Reference	Objective	AI Tool	Used Dataset(s)	Images Analyzed	Obtained Results
[18]	Classification of Diabetic Retinopathy	CNN and GNN	Kaggle (3464 images) and EyePACS dataset (35,000 images)	Fundus images	**Kaggle dataset:** Accuracy = 0.896; Kappa = 0.92; **EyePACS dataset:** Accuracy = 0.903; Sensitivity = 0.975; Specificity = 0.899
[19]	DR classification	Deep Graph Correlation Network (DGCN)	EyePACS dataset (1427 images) and Messidor dataset (1744 images)	Fundus images	**EyePACs dataset**: Accuracy = 99.01%; Sensitivity = 99.01%; Specificity = 98.43%; **Messidor dataset**: Accuracy = 99.60%; Sensitivity = 99.08%; Specificity = 100%
[20]	Multi-model domain adaptation for DR	CNN	DDR (12,522 images), IDRiD (516 images), Messidor (1200 images), Messidor-2 (1748 images), and APTOS-2019 (3662 images)	Fundus images	Accuracy = 90.6%; Sensitivity = 98.5%
[21]	Diabetic Retinopathy grading	GNN-CNN	APTOS2019 and Messidor-2	Fundus images	**APTOS2019**: Accuracy = 0.68; Kappa = 0.67; F1-Score = 0.66; **Messidor-2**: Accuracy = 0.85; Kappa = 0.90; F1-Score = 0.84
[22]	Diabetic retinopathy detection	CNN	7500 fundus eye images	Fundus images	Accuracy = 97%
[23]	Enhanced Diabetic Retinopathy detection	Semi-Supervised Graph Learning (SSGL)	Retinal images (3000 images)	Fundus images	Precision = 0.94; Recall = 0.93; F1-score = 0.96; Accuracy = 0.92
[13]	DR detection	CNN + BCNN (Bayesian deep learning)	APTOS 2019 and DDR	Fundus images	Accuracy = 0.92; Precision = 0.94; Recall = 0.93; F1-Score = 0.96
[14]	Diabetic Retinopathy Grading	CNN	APTOS2019 and Messidor-2	Fundus images	**APTOS2019**: Accuracy = 0.96; F1-Score = 0.94; Sensitivity = 0.94; Precision = 0.95; **Messidor-2**: Accuracy = 0.89; wF1 = 0.88; wKappa = 0.923
[15]	Diabetic Retinopathy detection and grading	CNN	APTOS and combination of EyePac + Messidor-2	Fundus images	**APTOS 5-class:** Accuracy = 82%; **APTOS 2-class:** Accuracy = 96%; **EyePac + Messidor-2:** Accuracy = 75%
[16]	Diabetic Retinopathy detection	CNN	388 retinal images	Fundus images	Accuracy = 0.99; Precision = 0.98; Sensitivity = 0.99; Specificity = 0.98
[17]	Diabetic Retinopathy classification	CNN + GNN	Messidor-2 + APTOS2019	Fundus images	**Messidor-2**: Accuracy = 0.97; Recall = 0.95; Precision = 0.96; Specificity = 0.99; **APTOS2019**: Accuracy = 0.98; Recall = 0.95; Precision = 0.968; Specificity = 0.99

**Table 3:** Summary of AI-based DME detection studies.

Reference	Objective	AI Tool	Used Dataset(s)	Images Analyzed	Obtained Results
[24]	DME classification	Multi-feature Decomposition Fusion Attention Network (MDFANet)	958 participants	Multicolor images (MCIs)	Accuracy = 95.4%; Precision = 94.2%; Recall = 92.2%; F1-score = 94.3%; Specificity = 95.6%; AUC = 95.1%
[25]	DME detection	Multilevel EfficientNetB0	UWF fundus imaging (UWF4DR) > 500	Fundus Images	AUROC = 98.20%; AUPRC = 96.99%; Sensitivity = 92.50%; Specificity = 95.77%
[26]	Morphologic DME pattern detection in OCT	Deep learning	Mendeley dataset and Department of Ophthalmology, Guangdong Provincial People’s Hospital, > 12,000 images	OCT images	**Diffused retinal thickening, cystoid macular edema, and serous retinal detachment:** Accuracy of 93.0%, 95.1%, and 98.8% respectively. Sensitivity of 93.5%, 94.5%, and 96.7% respectively. Specificity of 92.3%, 95.6%, and 99.3% respectively.
[27]	AMD/DME detection	CNN	Public dataset (45 images) and private dataset (934 images)	OCT images	Accuracy = 99.68%
[28]	Synthetic OCT B-Scan generation for DME	Generative Adversarial Networks (GANs)	Duke Eye dataset and Kermany dataset	OCT B-Scan images	**Original**: Precision = 0.62; Recall = 0.57; F1-Score = 0.52; Accuracy = 0.57; **Without-GAN-500**: Precision = 0.65; Recall = 0.65; F1-Score = 0.65; Accuracy = 0.65; **With-GAN-500**: Precision = 0.81; Recall = 0.70; F1-Score = 0.68; Accuracy = 0.70
[29]	DR/DME classification	Deep Convolutional Neural Network (CNN)	MESSIDOR (2072 images)	Fundus images	Accuracy = 97.91%; Sensitivity = 97.82%; Specificity = 98.64%; Precision = 0.97; F1-Score = 0.98
[30]	DME detection	Deep learning-based CenterNet Model	APTOS-2019 and IDRiD	Fundus images	**Aptos-2019 dataset**: Accuracy = 97.93%; **IDRiD dataset**: Accuracy = 98.10%

**Table 4:** Summary of AI-based drusen detection studies.

Reference	Objective	AI Tool	Used Dataset(s)	Images Analyzed	Obtained Results
[31]	3D drusen segmentation	Deep learning	OCT B-scans (1294 images)	OCT images	**Approach 2D-overall:** Accuracy = 0.47; Precision = 0.14; Recall = 0.12; **Approach 3D-overall:** Accuracy = 0.99; Precision = 0.67; Recall = 0.46
[32]	Exudate and drusen classification	SVM classifier	A dataset of 798 retinal images collected from various standard datasets, namely DIARETDB0, DIARETDB1, HEI-MED, STARE, and MESSIDOR	Fundus images	Sensitivity = 98.37%; Specificity = 99.64%; Accuracy = 99.67%
[33]	Recognition of drusen subtypes	Graph theory and deep learning	120 OCT images	OCT images	Accuracy = 98%; F1-score = 97%
[34]	Distinction between visible optic disc drusen and healthy optic discs	Residual Attention Network (RAN)	Optic disc drusen (116 images)	Optical Coherence Tomography–Angiography (OCT-A)	Accuracy > 98%

**Table 5:** Summary of AI-based CSR detection studies.

Reference	Objective	AI Tool	Used Dataset(s)	Images Analyzed	Obtained Results
[36]	CSC detection	InceptionV3 CNN	2504 fundus images (Eye-PACS) and verified by OCT	Fundus images	Accuracy = 85.7%
[37]	Diagnosis and classification of acute vs. chronic CSC	Custom CNN compared to VGG-16 and ResNet-50	2360 OCT images from 220 patients	SD-OCT images	CSC Diagnosis: Accuracy = 93.8%; AUROC = 0.989; Acute vs. Chronic: Accuracy = 97.6%, AUROC = 0.994
[38]	CSR Detection	Manual Augmentation, DarkNet, DenseNet	Public datasets (OCTID and fundus images)	OCT + fundus images	OCT: Accuracy = 99.78%; Fundus: Accuracy = 98.72%

**Table 6:** Summary of AI-based MH detection studies.

Reference	Objective	AI Tool/Method	Used Dataset(s)	Images Analyzed	Obtained Results
[40]	MH detection via edge-based and intensity segmentation	Hybrid method combining multilevel thresholding and derivation edge detection	SN Eye Hospital (200 images resized to 224 × 224)	SD-OCT images	Accuracy = 97.5%; Sensitivity = 88.3%
[41]	Classification of acute vs. chronic CSC using multiple OCT images	Deep learning with ResNet-50 + logistic regression ensemble (multi-scan decision module)	7425 images from 297 participants (Hangil Eye Hospital)	25 SD-OCT images per case	Accuracy = 94.2%

**Table 7:** Hyperparameters for the two datasets.

Hyperparameter	Values Explored	Selected Values
Learning rate	0.0005, 0.0002, 0.0001, 0.005, 0.002, 0.001, 0.01	0.0001
Number of epochs	1,2,…, 100	30
Batch size	16, 32, 64	32
Classes	-	7
Input Image Size	-	224 × 224
Optimizer	-	Adam (weight decay = 0.00001)

**Table 8:** Performance comparison of baseline models on the RFMiD dataset.

Model	Accuracy	Precision	Recall	F1-Score
AlexNet	0.632	0.443	0.392	0.407
ResNet50	0.671	0.536	0.438	0.462
ResNet101	0.684	0.649	0.430	0.493
MobileNet	0.710	0.679	0.459	0.518
GoogleNet	0.710	0.609	0.468	0.517
Vision Transformer	0.697	0.612	0.450	0.497
DenseNet121	0.703	0.597	0.472	0.515
**VisionTrack (ours)**	0.989	0.897	0.866	0.881

**Table 9:** Performance comparison of baseline models on the RetinalOCT Dataset.

Model	Accuracy	Precision	Recall	F1-Score
AlexNet	0.965	0.975	0.969	0.972
ResNet50	0.965	0.959	0.963	0.960
ResNet101	0.962	0.966	0.969	0.966
MobileNet	0.964	0.967	0.969	0.968
GoogleNet	0.960	0.962	0.968	0.965
Vision Transformer	0.968	0.965	0.969	0.967
DenseNet121	0.970	0.975	0.960	0.967
**VisionTrack (ours)**	0.980	0.979	0.978	0.979

**Table 10:** Comparison of VisionTrack with state-of-the-art methods.

Reference	Method(s)	Used Dataset(s)	Obtained Results
[6]	CoAtNet	RFMiD	Accuracy = 0.78
			Recall = 0.76
			Precision = 0.81
[44]	DFex-BeeHive Model	RFMiD	Accuracy = 0.987
			Sensitivity = 0.959
			Specificity = 0.997
[45]	CNN-RNN	OCT Dataset	Accuracy = 0.956
			Recall = 0.950
			F1 = 0.951
[46]	Deep sub-domain adaptation network with pseudo-label (DSAN-PL)	OCT Dataset	Accuracy = 0.965
			Recall = 0.965
			Precision = 0.961
[1]	Deep learning	RFMiD	Precision = 0.71
			Recall = 0.71
			F1-Score = 0.71
**Vision Track**	Multi-modal approach using CNN + GNN + LLM	RFMID	**Accuracy = 0.989**
			**Precision = 0.897**
			Recall = 0.866
			**F1-Score = 0.881**
			**Specificity = 0.999**
		RetinalOCT	**Accuracy = 0.980**
			**Precision = 0.979**
			**Recall = 0.978**
			**F1-Score = 0.979**
			**Specificity = 0.997**

**Table 11:** Paired *t*-test results comparing Multi-label Fundus against other models. Bold values indicate statistical significance (p<0.05).

Model	Accuracy (*p*)	Precision (*p*)	Recall (*p*)	F1-Score (*p*)
AlexNet	$2.1\times 10^{-16}$	$1.7\times 10^{-16}$	$1.9\times 10^{-16}$	$1.5\times 10^{-16}$
DenseNet121	$3.4\times 10^{-14}$	$2.8\times 10^{-13}$	$4.2\times 10^{-14}$	$5.1\times 10^{-14}$
GoogleNet	$7.2\times 10^{-16}$	$6.5\times 10^{-16}$	$8.3\times 10^{-16}$	$4.8\times 10^{-16}$
MobileNetV2	$1.1\times 10^{-15}$	$9.4\times 10^{-16}$	$5.7\times 10^{-16}$	$7.9\times 10^{-16}$
ResNet50	$1.5\times 10^{-15}$	$3.9\times 10^{-16}$	$3.1\times 10^{-16}$	$2.6\times 10^{-16}$
ResNet101	$5.3\times 10^{-16}$	$8.1\times 10^{-16}$	$6.4\times 10^{-16}$	$7.2\times 10^{-16}$
Vision Transformer	$9.6\times 10^{-16}$	$1.3\times 10^{-15}$	$1.1\times 10^{-15}$	$9.2\times 10^{-16}$

**Table 12:** Paired *t*-test results comparing Multi-label OCT against other models (epochs 1–30). Bold values indicate statistical significance (p<0.05).

Model	Accuracy (*p*)	Precision (*p*)	Recall (*p*)	F1-Score (*p*)
AlexNet	$1.7\times 10^{-15}$	$2.3\times 10^{-16}$	$1.9\times 10^{-16}$	$2.1\times 10^{-16}$
DenseNet121	$4.2\times 10^{-14}$	$3.1\times 10^{-13}$	$5.6\times 10^{-14}$	$6.4\times 10^{-14}$
GoogleNet	$8.5\times 10^{-16}$	$7.2\times 10^{-16}$	$9.1\times 10^{-16}$	$5.9\times 10^{-16}$
MobileNetV2	$1.3\times 10^{-15}$	$1.1\times 10^{-15}$	$6.8\times 10^{-16}$	$8.7\times 10^{-16}$
ResNet50	$1.8\times 10^{-15}$	$4.5\times 10^{-16}$	$4.0\times 10^{-16}$	$3.3\times 10^{-16}$
ResNet101	$6.2\times 10^{-16}$	$9.3\times 10^{-16}$	$7.5\times 10^{-16}$	$8.4\times 10^{-16}$
Vision Transformer	$1.1\times 10^{-15}$	$1.5\times 10^{-15}$	$1.3\times 10^{-15}$	$1.0\times 10^{-15}$

## Data Availability

Restrictions apply to the availability of these data. Data was obtained from third party and are available from the authorswith the permission of third party [42,43].

## Nomenclature

DR	Diabetic Retinopathy
AMD	Age-related Macular Degeneration
DME	Diabetic Macular Edema
CSR	Central Serous Retinopathy
MH	Macular Hole
CNN	Convolutional Neural Network
GNN	Graph Neural Network
LLM	Large Language Model
AI	Artificial Intelligence
RFMiD	Retinal Fundus Multi-Disease Image Dataset
OCT	Optical Coherence Tomography
WHO	World Health Organization
ViTs	Vision Transformers
LightGBM	Light Gradient Boosting Machine
HGB	Histogram-based Gradient Boosting
XGBoost	Extreme Gradient Boosting
RF	Random Forest
MLP	Multi-Layer Perceptron
DT	Decision Tree
LR	Logistic Regression
SVM	Support Vector Machine
KNN	K-Nearest Neighbor
GANs	Generative Adversarial Network
DGCN	Deep Graph Correlation Network
SSGL	Semi-Supervised Graph Learning
BCNN	Bayesian Convolutional Neural Network
CF	Color Fundus Photography
MDFANet	Multifeature Decomposition Fusion Attention Network
ICGA	Indocyanine Green Angiography
MCI	Multicolor Imags
UWF	Ultra-Widefield Fundus Imaging
